# Can packaging transparency influence consumer food waste behavior?

**DOI:** 10.1371/journal.pone.0329151

**Published:** 2025-08-05

**Authors:** Monireh Mahmoudi, Mina Mashayekhian

**Affiliations:** School of Packaging, College of Agriculture and Natural Resources, Michigan State University, East Lansing, Michigan, United States of America; Bina Nusantara University, INDONESIA

## Abstract

Consumer food waste remains a significant concern in the United States, where approximately 40% of food goes uneaten, leading to major economic and environmental consequences. Packaging plays a key role in food preservation and consumer decision-making, yet little research has examined how specific packaging features, such as transparency, influence consumer waste behaviors. To explore this, we conducted a three-week framed field experiment involving approximately 200 university students, focusing on deli meat—a perishable item commonly wasted in U.S. households. In this study, students were recruited to purchase pre-packaged deli meat from the research team. To simulate opaque packaging conditions, we applied white duct tape externally to otherwise transparent packages. Participants picked up their food on a self-selected date and consumed it over time. They were instructed to return any food scraps between one and three weeks later, based on when they personally deemed the food unappealing. Food scraps were measured to examine the link between packaging transparency and food waste. Observed patterns suggest that transparency may exert two contrasting influences on consumer behavior. On one hand, transparency may foster a greater sense of perceived control by providing visual cues that enhance consumers’ confidence in monitoring food quality and planning consumption, potentially leading to reduced waste. On the other hand, transparency may lead to overconfidence, reducing consumers’ vigilance in checking food quality. This illusion of ongoing freshness could decrease the frequency of quality checks and delay initial inspections, ultimately increasing the risk of spoilage and waste. While the results did not reach statistical significance, they consistently revealed directional trends, supporting an exploratory interpretation of the behavioral mechanisms involved. Although these findings are exploratory, they offer valuable insights into how packaging transparency may paradoxically shape consumer behaviors, emphasizing the importance of tailoring packaging strategies to mitigate food waste.

## Introduction

### Motivation

Consumer food waste represents a substantial challenge, constituting 40% to 50% of all food waste in the United States [64]. A key contributor to this issue is reliance on sensory cues—appearance, taste, smell, and texture—to judge edibility [35]. Foods that appear unappealing or past their perceived prime are more often discarded.

While packaging plays a vital role in preserving these cues, its influence on consumer food waste behaviors remains underexplored [26, 56]. This gap may stem from the diverse ways consumers handle packaging at home, complicating efforts to assess its effects. [2] and [3] identified several mishandling practices, including piercing the packaging to let it breathe, removing it entirely, repacking, and failing to use features like resealability as intended. Such practices can compromise packaging functionality, diminish protection, and accelerate spoilage, thereby increasing food waste [26].

To initiate an empirical study in this domain, we first selected a packaging feature through an extensive review of over 400 scholarly articles published since 2012 across fields such as Food Science, Environmental Science, Packaging Engineering, Supply Chain Management, Consumer Behavior, Marketing, Public Health, Nutrition, Sustainability Studies, and Economics. We focused on keywords including *consumer food waste*, *consumer food consumption*, *household food waste*, *household food consumption*, *food waste*, *packaging*, and *food packaging*. We also reviewed more than 100 industry articles examining the relationship between packaging and food waste reduction. A full account of this comprehensive review is presented in our recent scoping review paper (See [56]).

Our comprehensive review identified several key packaging features that could influence consumer food waste, including *Appealing Design and Graphics* for product appeal [22], *Advanced Shelf Life and Quality Indicators* to convey freshness [8, 19], *Unified and Readable Labels* for clearer communication [18], *Better Serving Tools* for easier use [26], *Multi-pack Options* for convenience [20], *Improved Opening and Closing* to preserve freshness [17], *Standard Date Labeling* for clarity [9, 21], *Sturdy Packaging* for transport protection [20], *Improved Resealing* to maintain freshness [10, 17], *Improved Dispensing* for usability [20], *Improved Handling* for ergonomics [20], *Transparent/Opaque Packaging* for visibility or privacy [11, 27], and *Variety of Sizes* to meet portion needs [12, 18]. Among these, *transparency*/*opacity* emerged as both a relatively impactful feature on consumer food waste (from the consumer’s perspective) [2, 3] and the least studied, making it a strong candidate for further investigation. To our knowledge, [27] is the only study that specifically examines packaging transparency, though it focuses on consumer food consumption behavior rather than food waste behavior. The recent review by [56] underscores this gap in the existing literature. The literature review also informed our choice of the food product: *deli meat*. The food selection process will be comprehensively discussed in Section “Materials and methods.”

Packaging transparency, like other packaging features, can play two contrasting roles in influencing consumer food waste behaviors. On one hand, it enhances product visibility, potentially reinforcing consumers’ perceived control—the belief in one’s ability to effectively manage outcomes [52]. Greater visibility can boost confidence in monitoring freshness and planning consumption. This heightened control may extend the period before perceived quality decline, reducing premature disposal and food waste [53]. In this study, we hypothesize that transparent packaging reduces consumer food waste by delaying perceived quality decline (Hypothesis 1). Transparency thus has the potential to support more thoughtful storage and consumption behaviors, promoting greater food utilization.

**Hypothesis 1.** *Packaging transparency extends the perceived quality retention period, potentially reducing food waste.*

On the other hand, behavioral economics highlights the risks of cognitive heuristics—mental shortcuts that simplify decisions but can lead to biased or suboptimal behaviors [54, 55]. While offering convenience, transparency may unintentionally foster overconfidence in perceived freshness, causing consumers to be less vigilant in monitoring product quality. Specifically, transparent packaging may reduce the frequency and delay the initiation of quality checks, weakening responsiveness to early signs of spoilage. This shift could potentially increase food waste by allowing unnoticed deterioration until corrective action is no longer possible. Accordingly, we hypothesize that transparent packaging increases consumer food waste by diminishing quality monitoring behaviors—reducing check frequency and delaying the first check (Hypothesis 2).

**Hypothesis 2.** *Packaging transparency reduces consumers’ food quality monitoring behavior (lower frequency and delayed onset of checks), potentially increasing food waste.*

To test the foregoing hypotheses, this study employed a framed field experiment by recruiting approximately 200 university students and monitoring their consumption and waste behaviors for deli meat over a three-week study period. Pre- and post-study surveys were conducted to capture a range of consumer behaviors.

To our knowledge, this study is the first empirical investigation of a specific packaging feature—transparency—within a longitudinal, real-world context. While prior diary-based studies have examined packaging characteristics such as date labeling, portion size, and ease of opening, they typically involve brief observation periods (e.g., one week), small household samples, and do not focus on specific food items. In contrast, our study evaluates one of the most commonly wasted food items in the U.S.—deli meat—over a longer three-week period. Methodologically, our research controls for demographic heterogeneity by using a homogeneous sample of university students, reducing confounds associated with age, income, household composition, and educational background. Lastly, we introduce and empirically measure three distinct consumer food monitoring behaviors for the first time: (1) number of quality checks between acquisition and disposal; (2) time from acquisition to initial check; and (3) time from acquisition to perceived quality decline. This research enables a detailed examination of how consumers interact with packaging transparency and how it affects food waste behavior.

### Background

#### Consumer food waste behavior.

Consumer food waste occurs across multiple stages of the household food journey, each influenced by distinct factors. In the pre-shopping phase, meal planning and shopping lists help prevent over-purchasing [42, 43]. Post-purchase, improper storage practices—such as incorrect fridge settings—can shorten shelf life [44, 45]. During preparation, poor culinary skills and cooking excessive portions worsen waste [46, 47]. In the consumption phase, food preference differences and poor leftover management contribute substantially [48, 49].

Household characteristics significantly influence waste generation. Larger households typically produce more waste overall, though per capita waste is often lower in families with children due to unpredictable eating habits [24, 34]. Situational factors such as living environment also matter; urban residents generally waste more food than rural counterparts, highlighting lifestyle and infrastructure impacts [32].

Psychological and emotional factors further shape food waste behaviors. Feelings of guilt can motivate waste reduction [39, 51], while food waste knowledge and personal sustainability involvement drive behavioral change [40, 41]. To enhance internal validity and cleanly test behavioral responses to packaging transparency, our study used a homogeneous student sample, minimizing confounds related to age, income, household composition, and education.

#### Consumer misunderstanding and misuse of packaging: An overlooked cause of food waste.

Consumers often overlook packaging’s role in reducing food waste. Packaging is rarely considered influential unless it directly inconveniences consumers [7]. Limited awareness of packaging formats, materials, and features further prevents consumers from optimizing food storage [6, 28]. Packaging serves multiple functions, including resealability, ease of handling, portion control, and protection against moisture, light, and gases [26]. Despite these benefits, packaging often carries negative perceptions. Consumers frequently associate unpackaged food with higher freshness and nutrition, preferring minimal packaging [30]. Environmental concerns reinforce these attitudes, with 90% of consumers believing packaging is more harmful than food waste [30]. However, life-cycle assessments show food waste has a much greater carbon footprint [30, 38].

Research on consumer handling of packaging after purchase is also limited [2, 3]. Actions vary widely, including keeping food in original packaging, piercing it, removing it entirely, repacking, or avoiding specific packaging types. Many consumers also fail to use packaging features (e.g., resealability) as intended. [30] reported that nearly half remove fresh food from original packaging shortly after purchase, undermining protective functions and accelerating food spoilage.

#### Packaging features and their potential to reduce food waste.

Food packaging acts as a crucial intermediary between food and consumers, influencing consumption behaviors and food waste outcomes. While prior research has examined food–consumer interactions [23] and food–packaging interactions [29], the role of consumer–packaging interaction in food waste remains underexplored [26].

Existing studies highlight several packaging features that may affect consumer consumption and waste outcomes. To avoid repetition, we omit the list of features here, as it appears in the former section. A recent survey by [3] of 1,000 U.S. consumers assessed the perceived importance of these packaging features in reducing food waste on a 5-point Likert scale. The highest-rated features were “maintain food freshness” (4.366), “food product dating” (4.122), “protection” (4.044), and “resealability” (3.901), followed by “size” (3.882), “use/cook direction” (3.655), “transparency” (3.641), “dispensing feature” (3.604), “safety direction” (3.604), “freshness indicator” (3.596), “easy to grip/shape” (3.440), “eco-friendly” (3.391), “material type” (3.363), “other statements” (3.105), and “graphics/illustrations/color” (2.539). Although transparency ranked moderately high, its direct impact on food waste remains underresearched. The only academic study on transparency, [27], focused on consumption, suggesting transparency can both promote consumption (salience effect) and reduce it through monitoring (monitoring effect). These insights emphasize the need to empirically measure how packaging transparency affects food waste and to further investigate the behavioral mechanisms involved.

## Materials and methods

### Ethical statement

All study procedures were reviewed and approved by the Michigan State University Institutional Review Board (IRB# STUDY00009968). In addition, supplementary surveys administered as part of the research were reviewed and approved by the Michigan State University Survey Committee. Student recruitment began on March 11, 2024, and concluded on March 25, 2024, through a university-wide email invitation. Informed consent was obtained electronically from all participants. The first page of the pre-study survey included a detailed consent form outlining the study’s purpose, procedures, and participant rights. Participants indicated their consent by clicking an “Agree” button before proceeding. The consent form clarified that participation was voluntary and that participants could skip any question, select “I don’t know” or “Other,” or withdraw at any time without penalty.

### Experiment duration and food selection

The first crucial step in our research design was selecting appropriate food items and determining the study duration. These decisions were closely linked, as food items needed to spoil within the experiment’s timeframe while the study had to last long enough to observe meaningful waste. Since the study was conducted on our university campus with student participants, it was also necessary to balance study length to maintain engagement without compromising the likelihood of waste. After careful consideration, we decided on a three-week field experiment.

**Fig 1:**
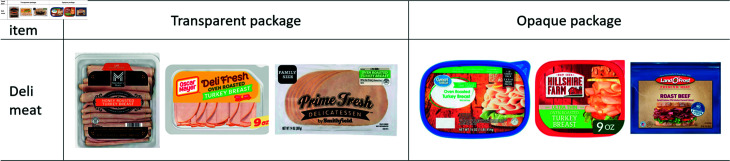
Transparent and opaque packaging for deli meat in the market.

We also developed a comprehensive set of criteria for selecting food items to maximize relevance, consistency, and measurable waste. The selected food items needed to be perishable, increasing the probability of waste during the three-week period. The selected food items also needed to be ones that consumers typically keep in their original packaging until consumption. This approach ensured uniform handling conditions and prevented the exclusion of packaging transparency as a variable during the experiment. Furthermore, the chosen products had to be available in both transparent and opaque packaging in the market, facilitating a thorough assessment of how packaging visibility impacts waste outcomes. Moreover, these items required minimal preparation and excluded extensive cooking, isolating the influence of packaging from variables like culinary skills. The food was also to be stored in a refrigerator or pantry, not a freezer, to reflect genuine perishability and waste patterns. Finally, we had to choose food items that are commonly and significantly wasted in U.S. households to minimize the risk of data scarcity. After thorough consideration, we selected *deli meat* as the food item that best met all the aforementioned criteria. Regarding the availability of food item in both transparent and opaque packaging, Fig 1 displays various brands offering this product in each packaging type. Regarding targeting commonly wasted foods, a survey conducted by OnePoll and HelloFresh involving over 2,000 U.S. participants identified deli meat as notably challenging food to consume before spoiling, with 16% of respondents respectively reporting difficulties [4].

### Experiment procedure

The study was designed to achieve two main goals: first, to provide causal evidence on how packaging transparency impacts consumer food waste; second, to incorporate supplementary pre- and post-study surveys to investigate underlying drivers of food waste behaviors. To support internal validity, the study utilized university students as participants, offering several advantages. The relatively uniform characteristics of the student population helped minimize variation in factors that show inconsistent relationships with food waste—such as age, income, education, and household size. For example, while some studies suggest older individuals generate less waste [24], others find no such effect [37]. Studies have found both positive and negative associations between household income and food waste. In some cases, higher income leads to increased waste due to over-purchasing or less sensitivity to food value [5, 33]. In contrast, in others, it is linked to lower waste due to greater awareness or better planning [31]. The effect of education on food waste is also mixed; some studies show that higher education levels are associated with reduced food waste due to increased environmental awareness [5, 33], while others suggest that highly educated consumers may waste more due to demanding freshness or trying new foods more frequently [32]. Household size also complicates interpretation, as larger households generate more total waste but less per capita, and those with children tend to waste more [34, 36]. By selecting a more homogeneous sample, we aimed to reduce the influence of these extraneous variables, enhancing the study’s practicality and clarity.

To maintain uniformity in the quality, quantity, and value of food, and to eliminate the burden on participants of finding specific brands or sizes, food procurement was directly handled by the research team. This approach differed from previous experiments where participants received various types of packaging for free (e.g., [27]). In this study, participants were required to buy food items from the research team, a crucial design choice since existing studies show that consumption and waste patterns differ when people purchase food themselves versus receiving it for free [50]. Selling food to students introduced limitations to the experimental setup. Specifically, the research team was not allowed to repackage food items to achieve desired packaging features (i.e., transferring food from original to alternative packaging). This limitation was enforced by the University Board of Trustees and food safety authorities, which prohibit repackaging food for sale on campus. As a result, packaging variation was constrained to using either the original packaging or modifying its external appearance.

**Fig 2:**
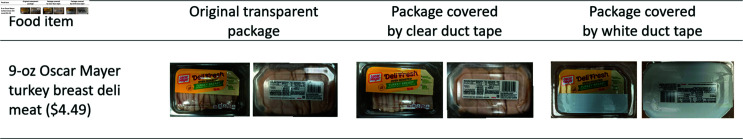
Package alternatives for deli meat.

The optimal approach for selecting a brand would involve choosing one that consistently provides the same quality, quantity, and value in both transparent and opaque packaging. However, the market lacked a brand meeting these criteria for deli meat; therefore, we altered the external surfaces of packages to achieve the desired packaging features. A Common brand was selected: 9 oz Oscar Mayer oven-roasted turkey breast for $4.49, originally packaged in transparent materials (Fig 2). To reduce novelty effects, it was important that the selected brand was readily accessible near the research site, making it likely that participants were already familiar with it. To ensure this, we conducted a thorough search of grocery stores within a 15-mile radius. Within this radius, there are 9 Meijer stores, 6 Kroger stores, and 3 Walmart stores selling the selected brands. Five other stores do not carry them. In other words, we found that 78% (18 out of 23) retail stores sold the selected brand.

To simulate opaque packaging, white duct tape was applied (Fig 2). Although this method controlled for product consistency, it introduced the alternative explanation that any observed effects might stem from the duct tape itself rather than packaging transparency. To address this potential confound, an additional group was created with packages covered in clear duct tape (Fig 2). This group served as an instrument-control, allowing us to isolate the impact of transparency. In summary, the design enabled comparisons of food waste across three conditions: opaque packages (modified with white duct tape), original transparent packages, and packages altered with clear duct tape. The experiment followed a between-subject design, and participants were randomized into the three groups.

Group A: Deli meat in original transparent packaging.

Group B: Deli meat covered with clear duct tape.

Group C: Deli meat covered with white duct tape.

To enhance realism and ensure public relevance, we used actual commercial deli meat products purchased from local retailers. Rather than using mock products, participants received real food items in their original sealed packaging. These packages were visually modified by covering them with either transparent or opaque white duct tape to simulate differences in packaging transparency. To ensure consistency across all experimental conditions, four undergraduate students were recruited and supervised by a graduate student. They were instructed to cut the duct tape to uniform dimensions and apply it consistently to each package, ensuring the appearance remained as homogeneous as possible across conditions. All modifications were pilot tested by sharing the prepared items with several colleagues unaffiliated with the study. We requested their verbal feedback on whether the taped packages resembled products they would typically encounter in a grocery store. Their confirmation that the packages appeared commercially realistic provided informal validation for the manipulation. This approach allowed us to evaluate behavior under realistic consumption conditions while avoiding potential safety or hygiene concerns. Using actual branded products in their sealed form helped ensure that the experimental manipulations were both believable and ecologically valid.

Student recruitment began on March 11, 2024 (about three weeks before the experiment) through a university-wide email. The email explained the study’s objectives and outlined clear eligibility criteria: being a current university student, regularly consuming the study’s food items, and agreeing not to share the food with others, such as friends or roommates. These conditions ensured that faculty and staff were excluded, participants were familiar with the food item, and the person responsible for food waste was the one consuming the food. The email also detailed participation steps, time commitment estimates, and the compensation participants could expect.

1. **Confirm Interest** (~1 min): Reply to the invitation email by March 25 (end date of the recruitment) to confirm participation.
2. **Pre-Study Tasks** (~10 min): (a) Complete the pre-study survey (b) Purchase the study food item (c) Schedule a pickup time
  Links for these tasks will be sent on March 26 and must be completed by March 29.
3. **Food Pickup** (~10–20 min): Pick up food on campus at your scheduled time between April 2–4.
4. **Return Leftovers** (~20–25 min): (a) Schedule a return time (April 8–26; link sent April 8) (b) Return leftovers in original packaging and complete the post-study survey
  A $50 Amazon gift card will be sent within 48 hours of survey completion.

The email also noted that the turkey breast deli meat is neither halal nor kosher. This information was included to ensure all potential participants were aware of these details before agreeing to join the study and purchase the food. To encourage honest reporting of food waste, students were assured that food scraps would be responsibly transported to and processed at the university’s composting center. To expand our reach further, we resent the invitation email on March 18.

One week before the experiment (on March 25), we sent a second email to reiterate the eligibility criteria and specific attributes of the study’s food items. We asked students to notify us within 24 hours if they were ineligible for any reason, ensuring the final participant roster was well-suited for the study. Qualified participants received a follow-up email on March 26 with detailed instructions, including links to the pre-study survey (Appendix S1), food item purchase, and pickup scheduling.

The first page of the pre-study survey included a consent form outlining the study and asked participants to click an “Agree" button to proceed. It also emphasized that participation was voluntary, and participants could skip questions, choose “I don’t know" or “Other," or withdraw at any time without penalty. The pre-study survey collected demographic data such as gender and age, assessed participants’ prior experience with food composting, and gathered information on typical consumption of deli meat. It also asked how often participants inspected the quality of this item post-purchase and how much was typically discarded due to spoilage. Additionally, the email outlined participation steps and emphasized food safety and freshness protocols. Specifically, it noted that deli meat should be refrigerated immediately, remain unopened for up to two weeks, and be consumed within 3–5 days once opened (see USDA guidance on lunch meat freshness).

To facilitate food ordering for participants and streamline distribution for the research team, a dedicated university-hosted website was created, allowing participants to place orders during a one-week period (March 26–31). Bulk purchases were made from a local retail store based on these orders. Procurement occurred from April 1–3, with food distributed the following day of each procurement to ensure freshness.

For efficient pickup and leftover drop-off, participants used the Doodle scheduling platform to book 2-hour time slots at their convenience. They were instructed to choose pickup times that allowed them to refrigerate deli meat within two hours of collection.

To maintain food quality, we rented a van to transport items directly from the store to campus, minimizing exposure to room temperature. A designated “store” area was established on campus, equipped with two refrigerators maintained at or below 41^∘^F to ensure freshness [13]. Thermometers continuously monitored temperature to comply with food safety standards and university regulations.

One day before their scheduled pickup, participants received a reminder email to collect their food from the on-campus store. Group A was notified on April 1, Group B on April 2, and Group C on April 3, with each group scheduled to pick up food the day after their reminder. On their designated day, participants collected their food items, and we provided white plastic bags to facilitate transportation and the return of any leftovers. This also helped minimize social desirability bias, reducing potential embarrassment or guilt about generating food waste. An undergraduate student, trained by the research team, provided verbal reminders of key study guidelines at pickup: consume the food naturally, maintain original packaging, and return leftovers in original packages, all placed in the provided white bags. Participants were also verbally assured that the food items—whether in their original transparent packaging, or visually modified with transparent or opaque white duct tape—were directly purchased from a local Meijer grocery store, had not been opened, and were safe for consumption. We emphasized that the duct tape was applied only to the external surface of sealed packages and did not compromise food safety. Additionally, participants were instructed not to remove the duct tape and to return any leftovers in the original package without making any alterations.

Returning leftover foods in their original packaging offered several advantages. First, it ensured participants did not remove food items from their packaging, preserving the study’s integrity in assessing the impact of package transparency on food waste. It also allowed uniform measurement of leftovers, including packaging weight, across all participants. Additionally, it facilitated verification of participants’ group names and corresponding food waste, preventing misattribution. Finally, keeping leftovers in original packaging helped contain unpleasant smells, minimizing discomfort for participants and the research team and reducing feelings of shame or embarrassment associated with returning leftovers.

One week after the initial pickup (on April 8), participants received an email with a Doodle link to schedule a convenient waste drop-off time within the next three weeks (April 8–26). April 26, 2024, was the end date of the study. Participants were instructed to return food packages once the items were consumed or deemed inedible. To ensure precise food waste measurement, a research assistant used a kitchen scale accurate to 1 gram to weigh all returned items. Food residues were collected daily for three weeks at the on-campus store and transported to the university composting center.

At their scheduled return time, participants completed a brief post-study survey (Appendix S2) to gather insights into their attitudes and beliefs about food quality. For the food item, participants reported how often they checked its quality during the study, the approximate date they felt compelled to check, and when the food became less appealing. The survey also collected feedback on participants’ experiences with the university’s composting service to control for other influencing factors.

**Fig 3:**
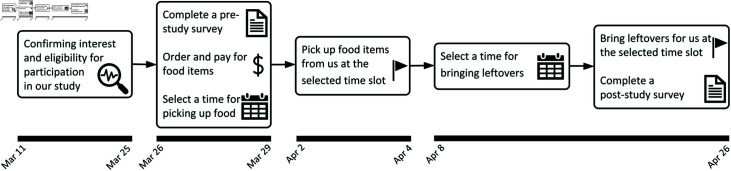
The study timeline.

To complete the study, participants were required to fill out the pre-study survey before picking up their food and the post-study survey upon returning their food package. Although participants were not obligated to answer every survey question—potentially leading to incomplete data—this approach aimed to improve data quality by reducing random responses submitted for the sake of completion. Reminders were sent during the final two weeks (April 15 and April 22) to ensure participants adhered to the timeline. Participants received a $50 gift card within 48 hours of returning their packages. To summarize, the study timeline is presented in Fig 3.

The dataset analyzed in this study was accessed for research purposes on April 26, 2024, the final date of data collection. During data collection, student names and email addresses were used solely for logistical coordination (e.g., survey distribution, package tracking, and compensation processing), and were stored in a secure, access-restricted file. For analysis and reporting, all data were de-identified to protect participant confidentiality. The authors did not retain access to personally identifiable information during or after the analysis phase, and all reported results are based on anonymized data.

## Results

A total of 225 students were initially recruited for the study. Upon review of survey completions and package return statuses, 203 participants successfully completed all study requirements. The primary reasons for exclusion were unreturned packages and non-compliance with experimental protocols. Specifically, some participants were disqualified for unopened packages, which suggested that the food had not been consumed at all. The sample size of approximately 200 university student participants, distributed evenly across three experimental conditions (65–70 per group), was determined and justified through a combination of theoretical novelty, logistical feasibility, and budgetary constraints.

First, to the best of our knowledge, no prior study has investigated the impact of any packaging feature—particularly packaging transparency—on consumer food waste behavior using a framed field experiment in a real-world setting. Very few studies have examined packaging features using longitudinal or diary-based designs. Existing work includes: [7], a one-week diary study of 61 Swedish households examining packaging date labeling, size, and ease of emptying for various food types; [37], a two-week diary of 380 Finnish households focused on packaging size and general food waste; [14], a 10-day diary of a single UK household studying date labeling and fresh produce waste; [15], a one-week diary of 17 Canadian individuals across 13 households focused on packaging size and date labeling (without specifying food types); and [18], a one-week diary of 37 Swedish households evaluating resealability, portion size, ease of opening/emptying, date labeling, and food safety information across categories. This literature provided no precedent or benchmarks to guide power calculations for our experimental setting. Our study represents the first empirical attempt to simulate consumer interaction with a packaging feature over three weeks, involving a substantially larger sample than existing diary-based studies.

Second, conducting a three-week field experiment with perishable food and 200 participants posed major logistical challenges. The research team managed procurement, packaging manipulation, and food distribution over three consecutive days, ensuring safe storage and product freshness. These tasks required tight coordination, staffing, and physical infrastructure.

Third, the sample size was shaped by a $17,000 research budget, which supported participant compensation, two home-style refrigerators, food procurement (later reimbursed), and labor costs for one graduate and four undergraduate assistants. These assistants handled packaging, distribution, weighing food scraps, and daily transport to the university composting center. Additional costs included duct tape, kitchen scales, van rental and fuel, and a hired driver for logistics. Given these constraints, we prioritized experimental control, real-world implementation, and data quality over increasing sample size.

**Fig 4:**
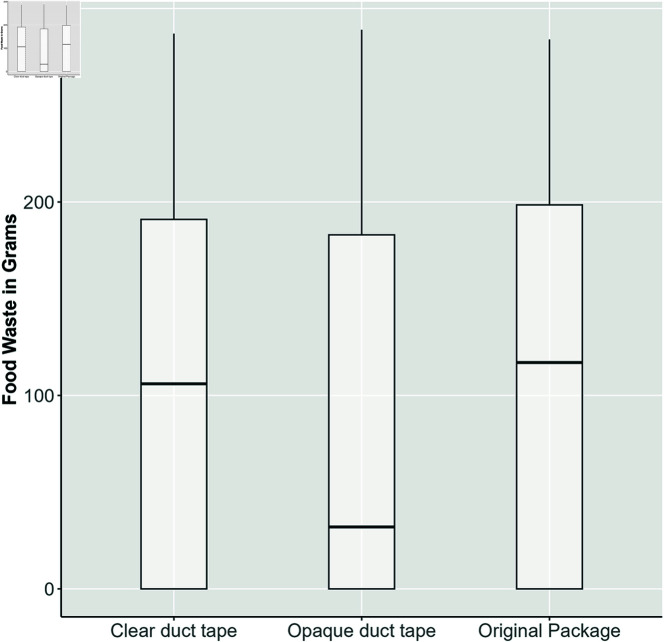
Box-plot comparison of packaging types for deli meat.

For deli meat, participants using the original packaging reported an average food waste of 114.84 grams (*sd* = 106.17). In contrast, participants in the clear duct tape and opaque duct tape conditions reported average waste of 106.94 grams (*sd* = 99.89) and 94.50 grams (*sd* = 105.31), respectively. No significant differences were observed between any two of the three groups (*p*>0.10). However, an interesting observation was the high percentage of participants reporting zero food waste for deli meat (48.50%). This can be explained by this fact that deli meats are often perceived as valuable or expensive, encouraging careful consumption and storage habits to avoid wastage. Fig 4 summarizes the field experiment results. Findings suggest that transparency appeared to negatively impact deli meat, possibly by giving consumers false confidence in freshness, leading to delayed consumption and increased waste.

These findings were further supported by ordinary least squares (OLS) regression analyses presented in Table 1, confirming the results even after controlling for additional variables [16]. Following [16], we present the results both with and without the control variables (Models 1-3). The list of control variables extracted from the pre-study survey is provided in Table 2. Although packaging transparency does not exhibit a statistically significant association with food waste in all models, the direction of the coefficients reveals a consistent pattern. The coefficients are consistently negative, pointing to a potential trend toward reduced waste under opaque packaging conditions. Although not statistically significant (except Model 1), the observed directional trend suggests that packaging transparency may potentially increase consumer food waste for deli meat, warranting further investigation.

**Table 1:** Ordinary Least Squares regressions of packaging types on food waste in grams.

Variable	Model 1	Model 2	Model 3
Clear duct tape	-7.385	-5.746	-9.248
	(17.793)	(17.905)	(17.986)
Opaque duct tape	-24.702^*^	-21.825	-21.885
	(17.509)	(17.983)	(17.965)
Food consumption control variable	No	Yes	Yes
Social demographic control variable	No	No	Yes
R^2^	0.010	0.043	0.095
Adj. R^2^	0.000	0.007	0.031
Num. obs.	203	198	198

Note: Regression results on food waste percentage for deli meat. The omitted reference group is the original (transparent) packaging. Standard errors in parentheses. Food consumption control variable includes the frequency of consumption, the frequency of quality check, the average percentage of food wasted, and the composting service. Social demographic control variables include age and gender. ${\;}^{***}p\;<\;0.01$, ${\;}^{**}p\;<\;0.05$, ${\;}^{*}p\;<\;0.1$

**Table 2:** Characteristics of the field experiment participants for deli meat in the original package, package covered by white duct tape, and package covered by clear duct tape groups.

		Original Package	White duct tape	Clear duct tape
		(N = 75)	(N = 65)	(N = 63)
Gender	Female	61.33 (46)	61.54 (40)	65.08 (41)
Age	18–24	86.67 (65)	81.54 (53)	88.89 (56)
	25–34	8.00 (6)	15.38 (10)	7.94 (5)
	35+	1.33 (1)	0 (0)	1.59 (1)
Composting Exp.	Yes/Maybe	18.67 (14)	21.54 (14)	25.40 (16)
Consumption Freq.	Daily/Several times a week	33.33 (25)	24.62 (16)	31.75 (20)
	Once a week	13.13 (10)	20.00 (13)	20.63 (13)
	Bi-weekly or Monthly	24.00 (18)	32.31 (21)	15.87 (10)
	Less freq. than monthly/Never	26.67 (20)	20.00 (13)	30.16 (19)
Freq. of Qual. Check	Average (Std.)	6.70 (5.85)	6.44 (4.50)	7.20 (6.26)
% of Discarded	Average (Std.)	19.88 (23.78)	18.73 (18.15)	16.50 (22.74)

Note: (i) All values are percentages (numbers) except for the frequency of quality check and percentage of discarded food, which has the average value (standard deviation); (ii) The totals for some columns may not equal 100% as participants were not forced to respond to every question.

To investigate the factors influencing food waste behaviors linked to food packaging transparency, post-study surveys were utilized to gauge participants’ perceptions and interactions with the food. Initially, participants were required to report the number of times they checked the food’s quality from the time of pickup to when they returned it. This figure was then averaged on a weekly basis to maintain uniformity across all participants. Next, participants noted the date when they first felt compelled to check the food’s quality. By comparing this date with the pickup date, the Initial Quality Check (IQC) Period was calculated—this is the number of days until participants felt the need to perform their first quality check. Furthermore, participants were asked to pinpoint the date when the food began to lose its appeal. The span between this date and the pickup date provided an estimate of the Perceived Quality Decline(PQD) Period—the duration the food was considered desirable.

As shown in Table 3, the use of opaque duct tape for deli meat packaging, relative to the transparent reference group, yields a statistically significant positive association with Quality Check (QC) frequency (${\;}^{*}p\;<\;0.1$). This indicates that participants performed QC more frequently when the product was packaged opaquely. Although the IQC and PQD period coefficients are not statistically significant, their negative directions may indicate a potential trend toward earlier initial quality checks and shorter perceived shelf life under opaque packaging conditions—offering exploratory insights that could be examined in future research.

**Table 3:** OLS regression of packaging types of deli meat on average weekly quality check (QC) frequency, initial quality check (IQC) period, and perceived quality decline (PQD) period.

	QC Frequency			IQC Period			PQD Period		
	Model 1	Model 2	Model 3	Model 1	Model 2	Model 3	Model 1	Model 2	Model 3
Clear duct tape	0.344	0.467	0.444	0.083	0.186	0.105	−0.206	−0.311	−0.089
	(0.334)	(0.334)	(0.339)	(0.992)	(1.023)	(1.031)	(1.020)	(1.046)	(1.051)
Opaque duct tape	0.457	0.599^*^	0.562^*^	−1.350	−1.267	−1.223	−0.714	−0.870	−0.730
	(0.327)	(0.327)	(0.334)	(0.970)	(1.002)	(1.015)	(0.997)	(1.025)	(1.034)
Food Consumption Control Variable	No	Yes	Yes	No	Yes	Yes	No	Yes	Yes
Social Demographic Control Variable	No	No	Yes	No	No	Yes	No	No	Yes
R^2^	0.011	0.093	0.102	0.014	0.036	0.063	0.003	0.035	0.069
Num. obs.	188	187	187	189	188	188	189	188	188

Note: Regression results on food waste grams of deli meat. The omitted reference group is the original transparent packaging. Standard error in parentheses. Food Consumption control variable includes the frequency of consumption, the frequency of quality check, the average percentage of food waste, all based on previous experience. It also includes the evaluation of how the participants evaluate the food composting service. Social Demographic control variables include age and gender. ${\;}^{***}p\;<\;0.01$; ${\;}^{**}p\;<\;0.05$; ${\;}^{*}p\;<\;0.1$.

To assess whether our sample size was adequate to detect meaningful behavioral effects, we conducted a post-hoc power analysis—a statistical method used to estimate the likelihood that a study would detect an effect if one truly exists. Assuming a moderate effect size (Cohen’s *d* = 0.4), which is commonly used in behavioral sciences [57], a two-tailed *t*-test with an alpha level of 0.05 and approximately 200 participants (distributed across three groups with ~65–70 per group) yields a statistical power of about 0.78. This meets the widely accepted standard of 0.80 for detecting medium-sized effects [58]. However, smaller effects (e.g., *d*<0.3) would have required a larger sample size to reach adequate power [59]. In simpler terms, our study had enough participants to detect moderate differences in behavior, but may have missed more subtle changes. This highlights the need for future replication studies with larger samples to confirm and expand on our findings.

## Discussion

Fig 4 and Table 1 collectively reveal a nuanced but consistent pattern: despite the absence of statistically significant differences across groups, the directionality of both descriptive and regression results suggests that packaging transparency may inadvertently contribute to greater deli meat waste. Specifically, the original (transparent) packaging condition exhibited the highest average waste, while the opaque condition showed the lowest. Although the differences are not significant at conventional thresholds, the negative coefficients across all OLS models (particularly Model 1) point to a potential behavioral mechanism in which transparency gives consumers false confidence in product freshness, leading to overextended storage and eventual spoilage. This insight aligns with existing literature suggesting that visual cues can sometimes mislead rather than inform consumer decision-making [16]. However, given the high proportion of participants reporting zero waste and the variability in individual consumption behavior, future research should explore whether this effect generalizes to broader consumer populations, different product types, or real-world purchase settings. Longitudinal studies or field experiments with tighter control over consumption environments may help clarify whether the observed directional trends translate into meaningful differences in household food waste outcomes over time.

The results presented in Table 3 also suggest that packaging opacity increases both the frequency of quality checks and prompts earlier initial inspections. In other words, rather than facilitating waste reduction, transparent packaging appears to reduce the frequency of quality checks and delay the timing of the initial inspection. These findings align with the behavioral mechanism discussed in the introduction section and support Hypothesis 2, which posited that the convenience and perceived freshness afforded by transparency may foster overconfidence, diminishing consumers’ vigilance in monitoring food quality. This behavioral pattern is consistent with cognitive ease [63], a heuristic in which easily processed or accessible information—such as visible product quality—reduces mental effort and fosters a false sense of security, thereby lowering consumers’ perceived urgency for active inspection or monitoring. As transparency reduces the perceived need for active quality monitoring, consumers may miss early signs of deterioration, ultimately increasing the risk of food spoilage and waste.

The regression results summarized in Table 3 also offer nuanced insights into Hypothesis 1, although none of the observed effects reach conventional levels of statistical significance. For deli meat, the estimated impact of packaging opacity is pronounced in direction, with a negative coefficient indicating that, in the absence of visual cues, consumers may perceive spoilage earlier and behave more cautiously. Although these results are not statistically significant, the directional patterns are consistent with theoretical expectations. As discussed in the introduction, packaging transparency can enhance perceived control over food freshness by offering visible cues that delay perceived quality decline [52, 53], which may, in turn, help reduce food waste.

Risk Perception Theory [1] also supports these observations in two ways: (1) consumers become more vigilant when visual information is absent, checking food quality more frequently and conducting their initial check sooner; and (2) they may feel more doubtful or uneasy about food quality when it is packaged in opaque materials. Together, these results illustrate the dual role of transparency: while it provides convenience and a sense of control, it may also foster cognitive shortcuts—such as reduced vigilance and delayed inspections—that suppress protective behaviors like quality monitoring. Taken together, while the findings should be interpreted with caution, they offer initial evidence that the behavioral benefits proposed in Hypothesis 1—namely, extending the perceived shelf life through enhanced perceived control—and the behavioral drawback proposed in Hypothesis 2—namely, the reduction of consumers’ food quality monitoring behavior—may be particularly relevant for high-risk food products. Our experiment focused exclusively on deli meat, a product with a relatively high risk of foodborne illness. It would be valuable for future research to examine whether similar or different effects of packaging transparency emerge for lower-risk foods, such as bread, particularly in relation to the PQD period.

Finally, while this study provides valuable insights into consumer behavior in response to packaging transparency, its scope is limited by the use of a university student sample and exclusive focus on deli meat. These constraints may restrict the generalizability of the findings to broader populations with varying food handling practices, cultural norms, and dietary preferences. Future research should replicate and extend these findings in more diverse household contexts and across a wider variety of food types—particularly those with different perishability levels, value perceptions, and packaging formats. Such efforts would enhance the external validity of the observed behavioral patterns and inform more comprehensive packaging strategies to mitigate food waste.

## Conclusion

Consumer food waste remains a pressing issue, with an estimated 40% of food in the United States going uneaten, resulting in significant economic and environmental costs. This waste primarily occurs when food’s sensory cues—taste, smell, and appearance—are no longer appealing. Packaging plays a critical role in preserving these cues. While many studies have examined technical food–packaging interactions to extend shelf life, few have explored how consumer interaction with packaging influences waste. Addressing this gap, our study focused on one impactful yet understudied feature: packaging transparency.

We recruited approximately 200 university students and monitored their consumption and waste over three weeks, focusing on a commonly wasted U.S. food item: deli meat. This perishable item was selected because it is typically stored in original packaging—allowing isolation of transparency effects—and is available in both transparent and opaque packaging, enabling direct comparison. It also requires minimal preparation, limiting confounds from cooking skills.

While our findings did not reveal a statistically significant relationship between packaging transparency and food waste, the consistent directionality observed across both descriptive statistics and regression models suggests a noteworthy pattern. Specifically, transparent packaging was associated with higher average waste, whereas opaque packaging corresponded with lower waste levels. These results point to a potential behavioral mechanism wherein visual access to a product may foster a false sense of freshness, leading to delayed consumption and increased spoilage. This interpretation aligns with prior research indicating that visual cues can sometimes misguide consumer judgment [16]. Although preliminary, this directional trend highlights an important area for further inquiry. Future research should investigate whether these effects persist across other food categories, consumer demographics, and real-world settings. Such studies could clarify the extent to which packaging design influences household food waste and inform more effective interventions in packaging strategy and policy.

Pre- and post-study surveys, along with regression analyses, provided further insight into the behavioral mechanisms underlying the observed waste patterns, despite the lack of statistically significant effects. Notably, opaque packaging was associated with more frequent and earlier quality checks, suggesting that reduced visibility may prompt consumers to monitor freshness more actively—potentially contributing to reduced waste. At the same time, opacity was associated with earlier perceived spoilage, suggesting that in the absence of visual cues, consumers may adopt more cautious judgments about food safety—potentially leading to premature disposal and increased food waste. While these findings are exploratory, they offer a useful foundation for future research. In particular, it would be valuable to examine whether similar mechanisms apply to lower-risk foods such as bread, especially around the perceived quality decline period, to better understand how packaging design can influence food waste across a broader range of contexts.

In summary, although statistical significance was not achieved, the directionally consistent results across models suggest that transparent packaging was associated with increased food waste and reduced monitoring behavior, while opaque packaging led to more frequent and earlier quality checks. Additionally, perceived quality decline occurred earlier for opaque packaging, indicating that reduced visibility may prompt consumers to judge freshness more conservatively. These patterns highlight the behavioral influence of packaging transparency, particularly for high-risk perishable items. For packaging designers and policymakers, these findings suggest that transparency alone may be insufficient to reduce food waste and may even backfire in some contexts. Incorporating additional freshness indicators—such as visual spoilage cues, color-changing sensors, or clearer date labeling—could help consumers make more informed decisions. For example, packaging that reveals mold development or changes in texture may prompt earlier detection of spoilage [60]. Colorimetric indicators can signal time-temperature abuse or spoilage [61]. Ambiguity in date labeling (e.g., “best by” vs. “use by”) contributes to premature disposal. Enhanced clarity may reduce unnecessary waste [62]. Balancing visibility with complementary signals of quality may be an effective strategy to support waste-reducing behaviors across diverse food types.

This study provides important insights into the behavioral effects of packaging transparency but also has some limitations. First, the use of a homogeneous student sample enhances internal control but restricts the generalizability of findings to broader populations with diverse food management practices. Second, the exclusive focus on deli meat—a single, high-risk, high-value perishable product—limits the applicability of results across other food types with different spoilage profiles, safety concerns, and price sensitivities. Third, while the use of duct tape to manipulate package transparency allowed for controlled comparisons, it may have unintentionally introduced perceptions related to hygiene or handling that could have influenced participant behavior. Fourth, the three-week study duration may not fully capture longer-term household food management behaviors or seasonal variations in consumption and waste. Fifth, although the study was designed to observe naturalistic consumer behavior, the structured instructions and scheduled prompts may have influenced participant routines, which should be acknowledged as a potential source of bias. Sixth, in real-world settings, consumers self-select packaging formats, which may reflect underlying preferences or behavioral tendencies. Our experimental design used randomized assignment to isolate the causal effects of packaging transparency, but did not capture these choice dynamics, which may limit external validity.

These limitations offer clear directions for future research. Expanding studies to include more diverse populations—across age groups, household types, and cultural contexts—can help evaluate whether the effects of transparency persist in real-world settings. Moreover, future work should explore how packaging transparency interacts with the perceived risk of foodborne illness and the monetary value of food. For instance, transparency may influence consumer food waste behavior differently for low-risk, low-cost items like bread versus high-risk, high-cost items like seafood or deli meat. To improve ecological validity, subsequent studies could avoid artificial manipulations—such as covering packages with duct tape—and instead use naturally available packaging formats or designs that are visually identical except for transparency. Additionally, longer-term diary studies or in-home trials without rigid prompting schedules could better capture habitual consumer behavior and reduce potential reactivity. Finally, future research should incorporate consumer choice into experimental designs to account for self-selection effects and preferences that may influence both packaging selection and food waste tendencies. Collectively, these research directions can support the design of evidence-based packaging interventions that better align consumer decision-making with sustainability goals.

## Supporting information

S1 Text (PDF)

S2 Text (CSV)
