# Supplementary material for: Can packaging transparency influence consumer food waste behavior?
Source: PLoS One. 2025 Aug 5;20(8):e0329151. doi: 10.1371/journal.pone.0329151 (PMC12324133; doi:10.1371/journal.pone.0329151)
Supplement: S1 Text — (PDF) [file pone.0329151.s001.pdf]

Supporting Information

S1. Pre-Study Survey

Q1. How do you describe yourself?
Male
Female
Non-binary / third gender
Prefer to self-describe: ______________________
Prefer not to say

Q2. How old are you?
Under 18
18–24 years old
25–34 years old
35–44 years old
45–54 years old
55–64 years old
65+ years old

Q3. Have you used a food composting service before?
No
Maybe
Yes

Q4. On average, how often do you consume deli meat?
Daily
Several times a week
Once a week
Bi-weekly or Monthly
Less frequent than monthly
Never

Q5. On average, how many times do you check the food quality of deli meat after buying it?
0 2 4 6 8 10 12 14 16 18 20

Q6. If any, on average, what percentage of deli meat do you dispose of because it is spoiled?
0% 10% 20% 30% 40% 50% 60% 70% 80% 90% 100%

S2. Post-Study Survey

Q1. Please rate your former experience with the food composting service at the university.
Extremely dissatisfied
Somewhat dissatisfied
Neither satisfied nor dissatisfied
Somewhat satisfied
Extremely satisfied

Q2. During the study period, how many times did you check the food

quality of deli meat?
0 2 4 6 8 10 12 14 16 18 20

Q3. During the study period, roughly on what date did you start feeling the need to check the food quality of deli meat to ensure it was not spoiled?
Date: ______________________

Q4. Did you find the deli meat less appealing at some point during the study period?
Date: ______________________
